# Functional Characterization of Gomisin N in High-Fat-Induced *Drosophila* Obesity Models

**DOI:** 10.3390/ijms21197209

**Published:** 2020-09-29

**Authors:** Joo Young Lee, Ji Hye Lee, Chong Kun Cheon

**Affiliations:** 1 Dental and Life Science Institute, Pusan National University, Yangsan 50612, Korea; prdm16@gmail.com; 2 Department of Life Science in Dentistry, School of Dentistry, Pusan National University, Yangsan 50612, Korea; 3 BK21Plus Project, School of Dentistry, Pusan National University, Yangsan 50612, Korea; 4 Department of Oral Pathology, School of Dentistry, Pusan National University, Yangsan 50612, Korea; 5 Research Institute for Convergence of biomedical science and technology, Pusan National University Yangsan Hospital, Yangsan 50612, Korea; 6 Department of Pediatrics, School of Medicine, Pusan National University, Yangsan 50612, Korea

**Keywords:** gomisin N, obesity, *Drosophila melanogaster*, endoplasmic reticulum stress response

## Abstract

Gomisin N (GN) is lignin derived from *Schisandra chinensis* that has been reported to exhibit hepato-protective, anti-cancer, and anti-inflammatory effects. However, its role in whole-body energetic homeostasis remains unclear. In this study, we employed *Drosophila melanogaster* as a diet-induced obese model to elucidate the effects of GN on lipid and glucose metabolism by measuring climbing activity, triglyceride levels, and lifespan under a rearing condition of a high-fat diet (HFD) containing 20% coconut oil, with or without GN. Constant exposure of flies to an HFD resulted in increased body weight and decreased climbing activity, along with a shortened life span. Importantly, the administration of GN to HFD groups lowered their body weight and induced a specific upregulation of lipid storage droplet (*Lsd*)*-2* and hormone-sensitive lipase (*Hsl*), in addition to improved lifespan. Importantly, GN in HFD groups appeared to downregulate heat shock protein Hsp90 family member (*dGRP94*), a key regulator of the endoplasmic reticulum stress response, which may also contribute to improved life span in the presence of GN. Taken together, these in vivo findings suggest that GN could serve as a useful agent for the prevention and treatment of obesity.

## 1. Introduction

The phenomenon of obesity is unique and distributed from childhood to old age. According to Organization for Economic Cooperation and Development (OECD) data, the adult obesity rate has increased steadily since 1990, with an average of 19.5%, led by the United States and Mexico at 38.2% and 32.4%, respectively, with lower rates of 3.7% and 5.3% for Japan and Korea, respectively [1]. In particular, 41.4% of obese people with a BMI over 30 died of cardiovascular disease, 9.5% of diabetes, and 4.7% of kidney disease [2]. Given both the risk of obesity-related health complications and the economic burden, reducing body weight is regarded as a major health benefit [3]. While anti-obesity drugs might be a promising solution to tackle obesity, the possible side effects or adverse drug reactions of these drugs are considered a public health concern [4]. The growing threat of obesity to global health and the undetermined efficacy, safety, and long-term effects of anti-obesity drugs has encouraged researchers to pay more attention and efforts to find an efficient and safe anti-obesity ingredient [5]. 

Gomisin N (GN) is a phytochemical lignan derived from the fruit of *Schisandra chinensis* (Chinese magnolia vine), a traditional herbal medicine available in some Asian countries. Previous studies have demonstrated the various pharmacological activities of *S. chinensis*, including anti-obesity, anti-cancer, anti-inflammatory, anti-oxidant, hepato-protective, and neuroprotective effects [6,7,8]. Recently, several reports have shown the involvement of GN in hepatic steatosis and adipocyte differentiation [9,10]. Jang et al. revealed that the high-fat diet (HFD)-fed obese mice displayed a higher total body and epididymal adipose tissue weight as well as elevated serum levels of glucose and triglycerides compared to normal diet-fed mice [9]. However, an intake of a high dose of GN was capable of reversing these trends [9], indicating that GN can prevent HFD-induced obesity and modulate the serum metabolic parameters. GN was also capable of significantly inhibiting the differentiation of 3T3-L1 preadipocytes at an early adipogenic stage through impairment of mitotic clonal expansion caused by cell cycle arrest at the G1/S phase transition [9]. Moreover, Yun et al. have recently demonstrated GN-dependent activation of the AMPK pathway, resulting in suppressed HFD-induced hepatic steatosis [10]. Despite recent efforts in deciphering molecular networks underlying the effects of GN on obesity in vitro, functional characterization of GN ultimately requires a systematic investigation in vivo. In this regard, obesity models developed in *Drosophila melanogaster* possess unique advantages with the ease of handling and genetic manipulations. In detail, *Drosophila* has tissues, organs, and systems analogous to all those involved in human obesity and associated metabolic diseases. Furthermore, *Drosophila* develops obesity and its related complications upon caloric overload, similar to humans. Moreover, most genetic networks involved in metabolic diseases are conserved between flies and humans, despite their anatomical differences [11]. For instance, there is a remarkable evolutionary conservation of lipolytic pathways and their components between mammals and insects. Just like the adipose triglyceride lipase (ATGL) knockout mice, homologous *brummer* (*bmm*) mutant flies are obese and impaired in acute lipid mobilization [12,13]. Similarly, the adipokinetic hormone receptor (AKHR) mutant flies become obese and are impaired in storage-fat mobilization. Furthermore, two perilipin family members called lipid storage droplet (*Lsd-1* and *Lsd-2)* have been demonstrated to be crucial for fat storage regulation in *Drosophila*, with *Lsd-2* as a specific marker for lipid accumulation [14,15]. Combining these findings, *Drosophila* poses itself as an ideal model in which the ever-expanding group of complications associated with obesity and metabolic disease can be studied with ease [16]. Herein, we provide another successful implementation of the *Drosophila* platform to demonstrate the anti-obesity effect of GN on metabolic dysregulation underlying these disorders. In this study, we investigated whether GN could prevent lipid and glucose metabolism and ameliorate endoplasmic reticulum (ER) stress response to exert therapeutic effects on an HFD-induced obese fly model. 

## 2. Results 

### 2.1. Feeding of Adult Drosophila with a High-Fat Diet Displayed Characteristic Phenotypes of Previously Established Obesity Models

*Drosophila melanogaster* has long served as an animal model system to investigate molecular mechanisms underlying metabolic syndromes, including obesity-related diabetes and cardiovascular diseases. With a high degree of homology between *Drosophila* and human orthologs, *Drosophila* can also be utilized as a valuable platform to examine the efficacy of novel drug candidates relevant to various metabolic syndromes. In this study, we sought to establish a diet-related obesity model in *Drosophila* to further investigate the molecular mechanisms underlying cellular and systemic responses to a variety of metabolic stresses [17]. 

**Figure 1:**
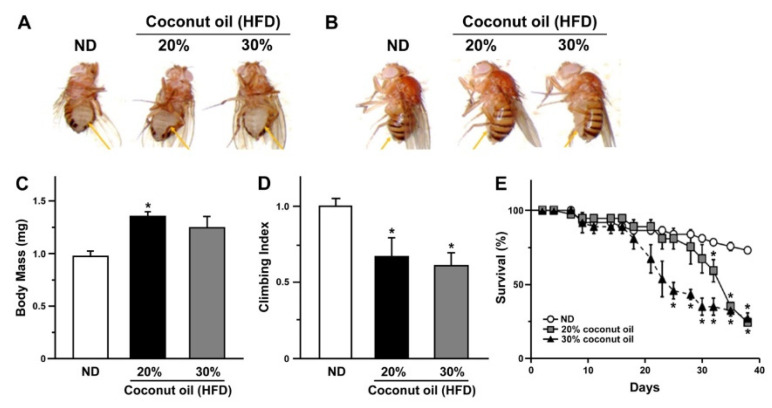
The effects of a high-fat diet on body size, body weight, climbing activity, and life span of adult flies. (**A**,**B**) The ventral view of flies (**A**) and lateral view of flies (**B**) are shown to compare the difference in body size and morphology between flies fed either with a normal diet (ND) or a high-fat diet (HFD) for 2 weeks (*n* = 12 per group).(**C**,**D**) The pooled data on body weight (**C**) and climbing index (**D**) of flies fed either with a normal diet (ND) or a high-fat diet (HFD) for 2 weeks are shown. Mean ± SEM are indicated. *, *p* < 0.05. (**E**) The life span of flies is shown for each rearing condition indicated (*n* = 12 per group). Mean ± SEM are indicated for experiments repeated three times. *, *p* < 0.05.

In an attempt to develop diet-induced obesity in adult flies, we fed flies with a regular corn meal-based media that was pre-mixed with or without 20 to 30% of coconut oil (*w*/*v*) (Figure 1A,B) [18,19]. When forced to grow under this high-fat diet (HFD) condition, the body mass of the HFD group fed with 20% of coconut oil was significantly increased compared to its control (Figure 1C). Consistent with previous reports [18,19], concurrent slowness was also evident when their climbing activity was monitored (Figure 1D). One of the hallmarks of flies on HFD includes a shortened life span [18,19]. Our finding also indicated a significant shortening of life span in HFD groups compared to the controls reared on a normal diet (Figure 1E), confirming a successful establishment of a diet-induced obesity model for further analyses.

### 2.2. A Treatment of GOMISIN N Reversed the Effect of a High-Fat Diet on Obese Phenotypes in Drosophila

**Figure 2:**
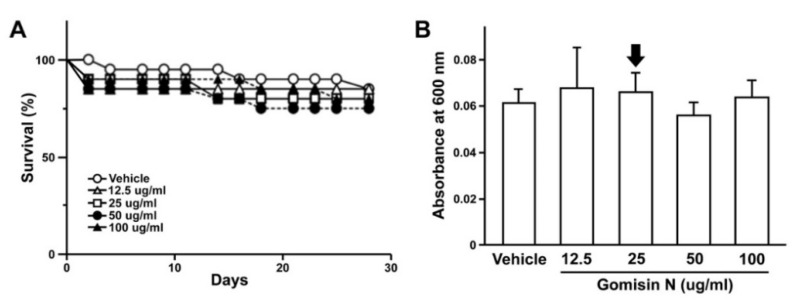
The effect of gomisin N on the survival of adult flies. (**A**) The viability of adult flies treated with gomisin N was measured by the survival rate. Flies were treated with various concentrations of gomisin N for up to 4 weeks (*n* = 12 per group). (**B**) The consumption of gomisin N was measured by monitoring the absorbance at 600 nm (OD_600_). Mean ± SEM are indicated for experiments repeated three times. The concentration selected for subsequent experiments is indicated with a black arrow.

Recent efforts in drug discovery that are relevant to obesity-related metabolic diseases have yielded a plethora of candidate molecules, many of which are often derivatives of natural compounds. In this study, we focused on one such molecule, gomisin N (GN), a lignan derived from the fruit of *Schisandra chinensis* with an anti-obesity effect [8]. To investigate whether GN could further modify the phenotypes of our obesity models in *Drosophila*, we first monitored the potential toxicity of GN to determine its optimal level to be applied for further analyses. As a previous study indicated the LC_50_ at a concentration of 0.125 μmol/mL (or 50 μg/mL) [20], we examined the survival rate of adult flies at a variable range of GN for 30 days. Interestingly, adult flies were capable of ingesting comparable amounts of growth media containing GN at a concentration higher than 50 μg/mL, without significant deterioration of survival (Figure 2). To minimize the potential toxic effect of GN, we applied a concentration of 25 (low dose) or 50 μg/mL (high dose) for subsequent experiments.

**Figure 3:**
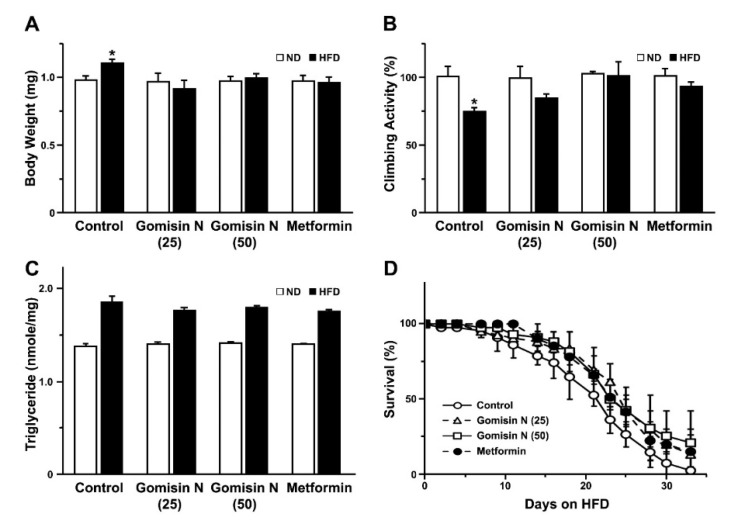
The effects of gomisin N on the HFD-induced phenotypes in adult flies. (**A**–**C**) The body weight (**A**), climbing activity (**B**), and levels of triglyceride (**C**) of adult flies fed either with an ND or HFD are shown for each rearing condition. Flies were treated with various concentrations of gomisin N or metformin for 2 weeks. (**D**) The life spans are shown for flies fed with an HFD with and without gomisin N or metformin. A total of 12 adult flies were included in each trial. Mean ± SEM are indicated for experiments repeated three times. *, *p* < 0.05.

When flies were exposed to GN for 2 weeks, a rearing condition with HFD failed to increase the total body weight (Figure 3A). Reduced climbing activity in HFD groups was also ameliorated by their exposure to GN (Figure 3B). Such reversal of HFD phenotypes was comparable to those grown in the presence of metformin, a well-established drug that is known to dampen the pathologic features of diabetic fly models [21] (Figure 3A–C). One of the key features of HFD fly models is an elevated level of triglyceride (TG) [19]. Our obese model indeed tended to increase the level of TG (Figure 3C). However, further administration of GN or metformin to HFD groups failed to significantly diminish the level of TG (Figure 3C). Meanwhile, the survival rate of HFD groups fed with gomisin N for an extended period was slightly improved in comparison with the vehicle control, indistinguishable from those reared with metformin (Figure 3D). These data together indicated the pharmacological effects of gomisin N equivalent to metformin on HFD-induced obesity in *Drosophila*.

**Figure 4:**
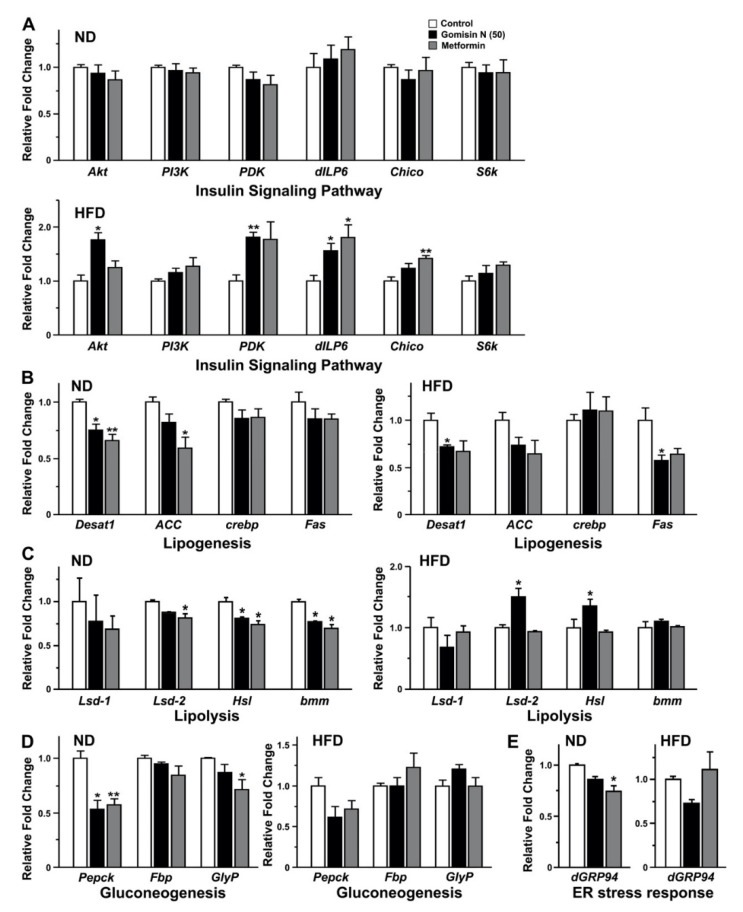
The effects of gomisin N on insulin signaling, lipogenesis, lipolysis, gluconeogenesis, and endoplasmic reticulum (ER) stress response in adult flies. The transcript levels of genes involved in insulin signaling (**A**), lipogenesis (**B**), lipolysis (**C**), gluconeogenesis (**D**), and ER stress response (**E**) are compared among adult flies fed with vehicle (control, white), gomisin N (50 μg/mL) (black), or metformin (10 mM) (gray). Comparisons of gene expression were performed with RT-qPCR experiments in triplicate. Mean ± SEM are indicated for experiments repeated three times. *, *p* < 0.05 and **, *p* < 0.01.

As HFD-induced obesity may also trigger alterations in the expression of genes involved in insulin signaling, lipogenesis, and gluconeogenesis [22], we sought to monitor the levels of representative transcripts relevant to these important metabolic processes. Importantly, GN was as effective as metformin in activating a subset of insulin signaling molecules under a HFD condition, including Akt1 (*Akt*) and insulin-like peptide 6 (*dILP6*) (Figure 4A). Moreover, some components of molecular networks engaged in lipogenesis, gluconeogenesis, and lipolysis, such as phosphoenolpyruvate carboxykinase (*Pepck*), desaturase 1 (*Desat1*), *Lsd-1*, *Lsd-2*, hormone-sensitive lipase (*Hsl*), and triacylglycerol lipase *brummer* (*bmm*), were also affected by both GN and metformin (Figure 4B–D). Interestingly, the suppressive effects of these drugs were evident even under a normal rearing condition (Figure 4B–D), suggesting a basal level of tonic activities maintained by the relevant molecular networks. Taken together, these data strongly support a pharmacological benefit of GN almost equivalent to metformin, a drug already acknowledged for its indication to target obesity-related type 2 diabetes.

### 2.3. Gomisin N Suppressed the Endoplasmic Reticulum Stress Response Induced by a High-Fat Diet in Drosophila 

It has been proposed that the endoplasmic reticulum (ER) stress response, or the unfolded protein response (UPR), could be activated under certain pathologic conditions, including obesity and type 2 diabetes [23]. For instance, prolonged ER stress responses may result in dysfunction and apoptosis of pancreatic β cells [24,25], thus aggravating the pathologic complications of obesity-related metabolic syndromes. As the molecular machinery controlling ER stress responses are largely conserved between *Drosophila* and human systems [26], we sought to investigate the effect of GN on ER stress responses in HFD flies. As an indicator of ER stress responses, we monitored the transcript level of *dGPR94*, a key component of ER chaperone systems that regulate the quality control of properly folded proteins [27]. The level of heat shock protein Hsp90 family member (*dGRP94*) mRNA in flies fed with a normal diet was affected by both GN and metformin to a greater extent (Figure 4E), suggesting the presence of tonic activity in the ER stress signaling pathway. Importantly, an administration of GN to HFD groups induced a noticeable decline in the level of dGRP94 transcripts, while the effect of metformin on its expression was inconclusive. These results thus indicate a potent pharmacological effect of GN on high-fat diet-induced ER stress signaling. 

## 3. Discussion

Obesity-related development of type 2 diabetes was once considered a pathologic condition that mostly concerned developed countries, but it has become a global health issue with a rising prevalence worldwide from 4.7% in 1980 to 8.5% in 2014 among adults over 18 years of age [28]. According to the WHO “*Global strategy on diet, physical activity and health*”, one of the beneficial approaches to tackle diabetes is to focus on population-wide approaches to facilitate a healthy diet, as well as physical activity, in an attempt to reduce a burden of obesity. In line with this effort, several chemical compounds mostly derived from natural ingredients have been synthesized in hopes of developing novel anti-obesity drugs, and their pharmacological activities have been examined in various experimental settings. In this study, we examined the effect of GN, a derivative from the fruit of *Schisandra chinensis*, on high-fat diet-induced obesity in *Drosophila melanogaster*. Our results successfully demonstrate a beneficial effect of GN on reversing the devastating effect of a high-fat diet on the viability of affected animals. Recent reports indicate the pharmacological effect of GN on the prevention of hepatic steatosis in high-fat diet-fed mice [10,29]. Here, we provide another layer of experimental evidence to support the anti-obesity effect of GN by utilizing *Drosophila*, a well-established in vivo animal model to study molecular mechanisms underlying various human disorders. *Drosophila* is widely accepted as a model to study obesity and relevant metabolic diseases and continues to provide valuable insights into our understanding of molecular pathology underlying these disorders [16,30]. Thus far, there have been limited numbers of studies performed in *Drosophila* to investigate the pharmacological benefits of phytochemicals derived from natural plants, with a potential link of isothiocyanates from radish sprouts to the regulation of PPARγ-coactivator 1α implicated in diabetes [31]. Here, we provide another successful implementation of the *Drosophila* platform for evaluation of novel phytochemicals in hopes of curing human metabolic disorders.

Our results indicate a suppressive effect of GN on lipogenesis, lipolysis, and gluconeogenesis (Figure 4B–D), presumably via ameliorating the ER stress response induced by a prolonged high-fat diet (Figure 4E). These findings are in line with previous reports demonstrating altered gene expression involved in lipogenesis and gluconeogenesis upon a treatment of GN in HepG2 cells as well as in obese mice [32]. Previous reports on similar experimental settings indicated GN-induced activation of AMPK in cultured cells as well as in mice [10,29]. In this study, we were unable to detect significant changes in the expression of AMPK (data not shown). However, a GN-induced upregulation of the Akt-pyruvate dehydrogenase kinase (PDK) signaling was evident in HFD groups (Figure 4A). Furthermore, our quantitative PCR analysis revealed a potential GN-induced upregulation of molecular players involved in insulin signaling under a HFD condition, including dILP6, a *Drosophila* insulin-like peptide corresponding to human insulin, and an insulin receptor substrate, Chico (Figure 4A). The expression of dILP6 was previously reported to respond to a HFD constituting 20% of coconut oil [33], equivalent to the growing condition in our study. A positive correlation between the expression of dILP6 and life spans has also been documented [34], consistent with our data indicating concurrent upregulation of dILP6 and extended life span in HFD groups fed with GN. Furthermore, Chico, a signaling molecule downstream of dILP6 [35], appeared to be concurrently upregulated in GN-treated groups, albeit with a lack of statistical significance (Figure 4A). Taken together, our data strongly suggest GN as a potential activator of insulin signaling upon exposure to a diet prone to obesity, thus posing it as an attractive drug candidate targeting type 2 diabetes.

**Figure 5:**
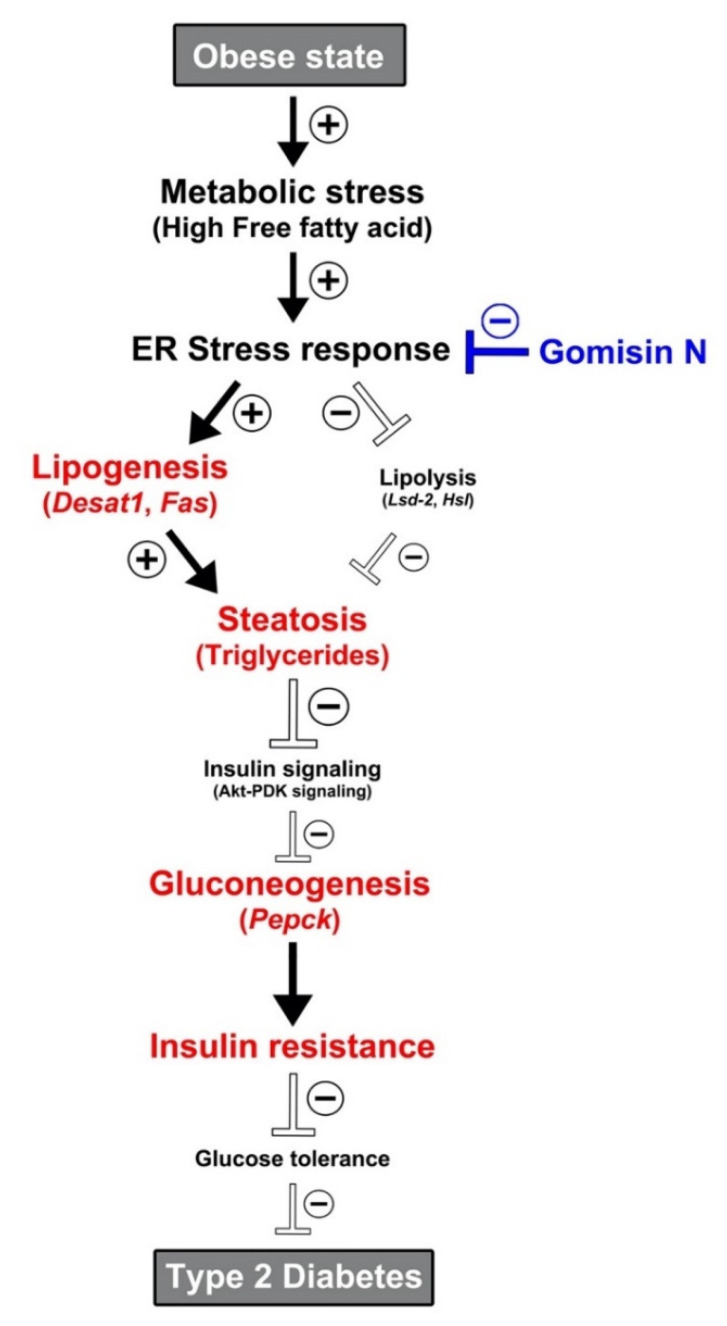
A schematic diagram describing altered metabolic processes in the obese state, along with potential regulation of ER stress response by gomisin N. The ⊕ and ⊖ symbols represent the activation and inhibition of subsequent metabolic pathways, respectively.

In conclusion, we present in vivo evidence supporting the pharmacological potential of GN as a novel drug candidate to target obesity-related type 2 diabetes via suppression of ER stress response (see also Figure 5 for the summary). By adopting *Drosophila melanogaster* as an animal model to study HFD-induced obesity, we provide an insight into molecular mechanisms underlying the beneficial effects of GN against obesity-related type 2 diabetes. 

## 4. Materials and Methods

### 4.1. Reagents and High-Fat Diet Preparation

Gomisin N (GN) was obtained from Chemfaces (Wuhan, China). Antimycin A was purchased from Sigma-Aldrich (St. Louis, MO, USA). Growing media containing high levels of fat were made from a standard laboratory fly food, which was replenished with 20% or 30% coconut oil in weight to volume ratio as a source for increased saturated fat in the diet. As there were no prominent phenotypic differences under these conditions (see Figure 1), the results obtained from flies reared on a diet containing 20% coconut oil were reported throughout the study.

### 4.2. Fly Rearing and Feeding Assay

The *w^1118^* flies were obtained from Bloomington Drosophila Stock Center and reared on standard cornmeal medium in an incubator at 25 °C and 40% humidity. The virgin *w^1118^* female flies were collected for 0 to 2 days after eclosion and used in all experiments. For a life-span analysis, flies were kept on standard food for 2 or 3 more days, equivalent to 3 to 5 days after eclosion, and placed in a plastic vial containing standard food. A filter paper was placed in this vial, which was soaked with 100 mM of sucrose, 0.04% of bromophenol blue, and GN at specified concentrations. The intake of GN was estimated by the absorbance of the dye (optical density (OD) value at 600 nm). Following the rearing in this vial for 24 h, all flies showed apparent blue coloration in their abdomen and were frozen at −80 °C for further OD measurements. Briefly, 20 flies were homogenized in 500 µL of phosphate-buffered saline (PBS) with the subsequent refrigerated centrifugation at 14,000× *g* for 5 min. The supernatants were mixed with 100 µL of PBS and centrifuged again under the same condition to further remove remaining debris. The absorbance of blue dye in the supernatant was measured at 600 nm using a Synergy HTX Multi-Mode Reader (BioTek Instruments, Winooski, VT, USA). To determine the toxicity of GN, we monitored the survival of 20 flies for 4 weeks in each run. Once placed in a vial, flies were transferred to a fresh vial containing a filter paper soaked with GN every other day. The survival rate was estimated by counting the number of surviving flies and by dividing it with 20 at each time point.

### 4.3. Measurement of Climbing Ability 

The viability of flies under different rearing conditions was evaluated using a climbing assay, in which the climbing index as a percentage was calculated. For each cohort, we kept 20 flies on day 14 in an empty vial for 1 h at room temperature for environmental adaptation. After tapping each vial onto the benchtop, we took a photograph for up to 8 s. This procedure was repeated 4 times in series. Captured images were analyzed by counting the number of flies residing in individual vertical quadrants of each vial at a specified time. The flies remaining in the highest quadrant were given 4 points, with a sequential 1-point deduction in the following quadrants. The 0 point was given to the flies remaining at the bottom of each vial for up to 8 s. The climbing score was recorded for each vial by adding all points given to flies in individual vials. The average climbing score was obtained by 3 independent tests and normalized to the score obtained from the groups rearing under a normal diet condition. 

### 4.4. Measurement of Triglyceride (TG)

TG concentration was estimated by using a reagent TAG (triacylglycerol) assay kit (Biomax, Seoul, Korea) following the manufacturer’s protocol. TG assay was performed using whole-body homogenization on the 14th day of GN treatments. Three flies were homogenized in 600 µL of PBT (PBS 1×, 0.05% Triton X-100), with the subsequent centrifugation at 4000× *g* and 4 °C for 15 min. The supernatant after homogenization was heated at 70 °C for 10 min. Then, 20 µL of the supernatants was added to 200 µL of the TAG reagent. The mixture was incubated at 37 °C for 10 min. The absorbance was read at 550 nm using a Synergy HTX Multi-Mode Reader (BioTek Instruments, Winooski, VT, USA).

### 4.5. Measurement of Life Span

The effects of GN on the life span of flies were evaluated under different rearing conditions. Twenty virgin females at days 3 to 5 were collected using CO_2_-induced anesthetization and placed in standard or high-fat food in the presence or absence of GN. The survival rate was estimated as described above. Since the melting point of coconut oil is relatively low, we kept all flies at 21 °C and 40% humidity.

### 4.6. Real-Time Quantitative PCR Analysis

Real-time quantitative PCR experiments were performed on the 14th day of GN treatments. Moloney murine leukemia virus reverse transcriptase (Invitrogen, Carlsbad, CA, USA) was used to reverse-transcribe total RNA extracted from whole body homogenization of flies, according to the manufacturer’s instructions. Subsequent quantitative PCR analyses were performed using a fluorescence temperature cycler (Applied Biosystems, London, UK). The primer sets used in this study are listed in Table S1. The relative gene expression levels were calculated by normalization of the transcript levels of target gene to that of actin.

### 4.7. Statistical Analysis

Data of two experimental groups were compared using unpaired two-tailed Student’s *t*-tests. Multiple comparisons were carried out using one-way analysis of variance (ANOVA), followed by Tukey’s post-hoc tests. Differences were considered significant when *p* was < 0.05.

## Supplementary Materials

Supplementary materials can be found at https://www.mdpi.com/1422-0067/21/19/7209/s1. Table S1: The primer sets used in this study.

## Abbreviations

Akt	Akt1
ATGL	adipose triglyceride lipase
Bmm	triacylglycerol lipase brummer
Chico	chico
Crebp	cyclic-AMP response element binding protein A
Desat1	desaturase 1
dGRP94	heat shock protein Hsp90 family member
dILP6	insulin-like peptide 6
Fas	fatty acid synthase 1
FBP	fructose 1, 6-biphosphatase
GAPDH	glyceraldehyde-3-phosphate dehydrogenase
GLYP	glycogen phosphorylase
Hsl	hormone-sensitive lipase
LSD-1	lipid storage droplet-1
LSD-2	lipid storage droplet-2
PDK	pyruvate dehydrogenase kinase
PEPCK	phosphoenolpyruvate carboxykinase
PI3K	Pi3K21B
S6k	ribosomal protein S6 kinase
