# Supplementary material for: Functional Characterization of Gomisin N in High-Fat-Induced Drosophila Obesity Models
Source: Int J Mol Sci. 2020 Sep 29;21(19):7209. doi: 10.3390/ijms21197209 (PMC7582321; doi:10.3390/ijms21197209)
Supplement: Supplementary file 1 [file ijms-21-07209-s001.pdf]

Supplementary Materials:

Table S1: The primer sets used in this study.

Gene	Forward	Reverse	Association No.
Akt	AAGAGGGGCGAGCACATAAAG	GTAACCCATCAGTCTTCCATCG	FBgn0010379
Pi3k	AATCTGCCTGTTGCCCAATG	ATAGCCCAGTGGCATCTGTTT	FBgn0015279
PDK	TGCTTAGTGCAGAATTAGG	GGCATCGTTCAGGTCGAAAG	FBgn0020386
diLP6	GTCCAAAGTCCTGCTAGTCCT	TCTGTTCGTATTCCGTGGGTG	FBgn0044047
chico	ACATCAATCGCCGTTTGGACA	GAGAACGATGCCGAATCCAC	FBgn0024248
S6k	ACTGGGCGCTCTCATGTTTG	TTGGCTTTCAGAATGGTCT	FBgn0015806
Pepck	GAACTGACGGACTCTGCTTAC	GGTGCGTTCGGGATCACAA	FBgn0003067
fbp	CCGCCATCAAGGCTACATCAT	TTGGAGAGCACGTCCAGTTTC	FBgn0032820
GlyP	TCCACCCTGAGGGACTACTAC	GGTGTTGGTCAGTGAGCGAC	FBgn0004507
desat1	CCGGAGTGCTCTTCGAGTG	CAGCCAGATGGAGGTAACCG	FBgn0086687
Acc	GACCTCGCAGCTTAAAAGTAACG	GCATGGTTCTTCATCTTGTCGT	FBgn0026620
crebA	AAAGATCCCACCGTCATACTCA	CCGTCTTGATCGGCTGTGT	FBgn0004396
Fas	TGACCAACAGTTCTTCGGTGT	GCGTCAATAATAGCTTCATGGGT	FBgn0027571
Plin1	TCAAGCAAATCGGTAATGCTGT	CCGACTGTGAAAGCGCCAT	FBgn0039114
Plin2	ATTGCCCGTGGTAAATGCG	CGAAGACACGATTTTTGCCTTT	FBgn0030608
HSL	CTGGAGGCGACCTATGGAAC	GCTCGTCAAAATCGTACTCGTG	FBgn0034491
Bmm	GTCTCCTCTGCGATTTGCCAT	CTGAAGGGACCCAGGGAGTA	FBgn0036449
dGrap94	CGGAGGTCAACCGCATGAT	CAGAGCCAATAGGCGGATCTT	FBgn0039562
